# Assessment of cochlear synaptopathy by electrocochleography to low frequencies in a preclinical model and human subjects

**DOI:** 10.3389/fneur.2023.1104574

**Published:** 2023-07-07

**Authors:** Raymond A. Haggerty, Kendall A. Hutson, William J. Riggs, Kevin D. Brown, Harold C. Pillsbury, Oliver F. Adunka, Craig A. Buchman, Douglas C. Fitzpatrick

**Affiliations:** ^1^Department of Otolaryngology, University of North Carolina at Chapel Hill, Chapel Hill, NC, United States; ^2^Department of Otolaryngology, The Ohio State University, Columbus, OH, United States; ^3^University of North Carolina School of Medicine, Chapel Hill, NC, United States; ^4^Department of Otolaryngology, Washington University in St. Louis, St. Louis, MO, United States

**Keywords:** electrocochleography, auditory nerve, hair cells, cochlear microphonic, deep learning

## Abstract

Cochlear synaptopathy is the loss of synapses between the inner hair cells and the auditory nerve despite survival of sensory hair cells. The findings of extensive cochlear synaptopathy in animals after moderate noise exposures challenged the long-held view that hair cells are the cochlear elements most sensitive to insults that lead to hearing loss. However, cochlear synaptopathy has been difficult to identify in humans. We applied novel algorithms to determine hair cell and neural contributions to electrocochleographic (ECochG) recordings from the round window of animal and human subjects. Gerbils with normal hearing provided training and test sets for a deep learning algorithm to detect the presence of neural responses to low frequency sounds, and an analytic model was used to quantify the proportion of neural and hair cell contributions to the ECochG response. The capacity to detect cochlear synaptopathy was validated in normal hearing and noise-exposed animals by using neurotoxins to reduce or eliminate the neural contributions. When the analytical methods were applied to human surgical subjects with access to the round window, the neural contribution resembled the partial cochlear synaptopathy present after neurotoxin application in animals. This result demonstrates the presence of viable hair cells not connected to auditory nerve fibers in human subjects with substantial hearing loss and indicates that efforts to regenerate nerve fibers may find a ready cochlear substrate for innervation and resumption of function.

## Introduction

Recent animal studies have shown that synapses between the inner hair cells and the auditory nerve, rather than hair cells themselves, are the elements most sensitive to destruction by moderate noise exposure (1–4). Using noise exposures that produced only temporary threshold shifts and no loss of hair cells, up to half of the synapses can be lost, despite thresholds for distortion product otoacoustic emissions, auditory brainstem responses, and compound action potentials returning to normal. The explanation is that excitotoxic effects of over-stimulation is greatest in fibers with low spontaneous rates that have high thresholds, while the high-spontaneous rate fibers that have low-thresholds remained intact (5–7).

Since determination of audiometric thresholds are the primary basis for detecting human hearing loss, and thresholds would be unchanged if the fibers with the lowest thresholds remain intact, the clinical implications of a large but undetected loss of auditory nerve fibers are obvious. Consequently, a substantial effort has been mounted to determine if cochlear synaptopathy is present in humans and if it leads to ‘hidden hearing loss,’ i.e., deficits such as decreased ability for hearing in noisy backgrounds that are not detectable by a change in the audiogram. In general, this effort has shown that cochlear synaptopathy in humans occurs anatomically primarily as a function of age (8–10), but has not conclusively shown a functional correlate [reviewed by (11)]. One approach has been to test different audiometrically-normal populations that are expected to have higher or lower levels of noise exposure (12, 13), or that report greater lifetime noise exposures (14–16). In general, these studies have found no performance decrements with greater noise exposure on a variety of primarily speech in noise perception tests that, theoretically, should be affected by cochlear synaptopathy. Objective tests, including amplitude and latency of waves I and V (13, 17, 18), middle ear muscle reflex (19, 20), the summating potential (SP), or SP to compound action potential (CAP) ratio in electrocochleography (ECochG) (19, 21), and envelope and frequency following responses (22) have also yielded mixed results, with some showing important suggestive evidence of increased cochlear synaptopathy in groups with greater noise exposure.

More recent animal work (7, 23–25) as well as older studies (26, 27) suggest that the loss of synapses may be partially reversible, and that the excitotoxic effects may include low spontaneous rate fibers as well (28). Recovery of synaptic function could explain why the effects of cochlear synaptopathy in noise-exposed but relatively young human subjects have been difficult to show. However, in older subjects with permanent threshold increases the effects of cochlear synaptopathy may be present. Anatomical studies of immunolabeled synapses and fiber counts in the osseous spiral lamina on human temporal bones suggest that synaptopathy is present and increases with age (8–10). Another finding consistent with cochlear synaptopathy under a condition of substantial hearing loss is a high correlation reported between ECochG amplitude and speech perception outcomes in adults with cochlear implants (CIs, *r* = 0.69) (29–31). Because the relationship between preoperative audiometric thresholds and postoperative speech perception outcomes is weak (32–34), the ECochG measurement must be capturing information about the health of spiral ganglion cells available for electrical stimulation that is different from the audiogram. The explanation offered by Fontenot et al. (29) is that hair cell activity recorded from ECochG is disconnected from nerve fibers, i.e., cochlear synaptopathy is present. In this view, hair cell activity acts as a metric of ‘cochlear health’, in that hair cells within a functional organ of Corti can help support spiral ganglion cells and thus lead to better speech perception outcomes.

To test this view, we created cochlear synaptopathy in gerbils using neurotoxins and characterized the ECochG for neural and hair cells in normal hearing gerbils and in gerbils with a high frequency noise exposure intended to mimic the sloping pattern of hearing loss found in many adult CI subjects. The sloping pattern consists of little or no sensitivity to high frequencies (greater than about 1,500 Hz) and variable hearing to low frequencies, including some with minimal or moderate hearing loss (35). The responses to the gerbils before and after neurotoxins were then compared to ECochG recordings from human CI subjects and others where the round window (RW) was available during surgery. We report that the human groups displayed proportions of hair cell and neural activity in their ECochG recordings to low frequencies similar to the recordings of animals treated with neurotoxins to produce cochlear synaptopathy.

## Materials and methods

### Animal and human subjects

Protocols for the use of gerbils, *Meriones unguiculatus*, were approved by the Institutional Animal Care and Use Committee (IACUC) at the University of North Carolina at Chapel Hill, following the standards of the National Institutes of Health and Committee on Care and Use of Laboratory Animals.

Data from human subjects was obtained intraoperatively with approval of the Institutional Review Boards at the University of North Carolina at Chapel Hill and the Ohio State University. Informed consent was obtained from all adult participants. Parental consent was obtained for pediatric subjects and patient assent was obtained from children between 7 and 18 years. Inclusion criteria for ECochG were that potential subjects were scheduled to receive a CI after the medical and audiological evaluation had established candidacy or were undergoing a surgery where the RW was accessible. Potential candidates were excluded from the study if they were not fluent in English, were undergoing revision surgery, or presented with severe inner ear malformations. The subject pool was therefore a mix of subjects of all ages typically seen at large centers for otologic or neurologic surgeries.

### Experimental design

#### ECochG principles

The ECochG response contains contributions from hair cells and the auditory nerve that mix in complex ways as stimulus frequency and intensity are varied. A main component from hair cells is the cochlear microphonic (CM), so-called because it faithfully mirrors the input waveform to the point that a listener can understand what was said when listening to the ECochG recording (36, 37). To low frequencies, the CM is mixed with the auditory nerve neurophonic (ANN), a neural component that also follows the stimulus waveform due to phase-locking in auditory nerve fibers (38, 39). Thus, to low frequencies the CM and ANN are mixed in ECochG.

**Figure 1:**
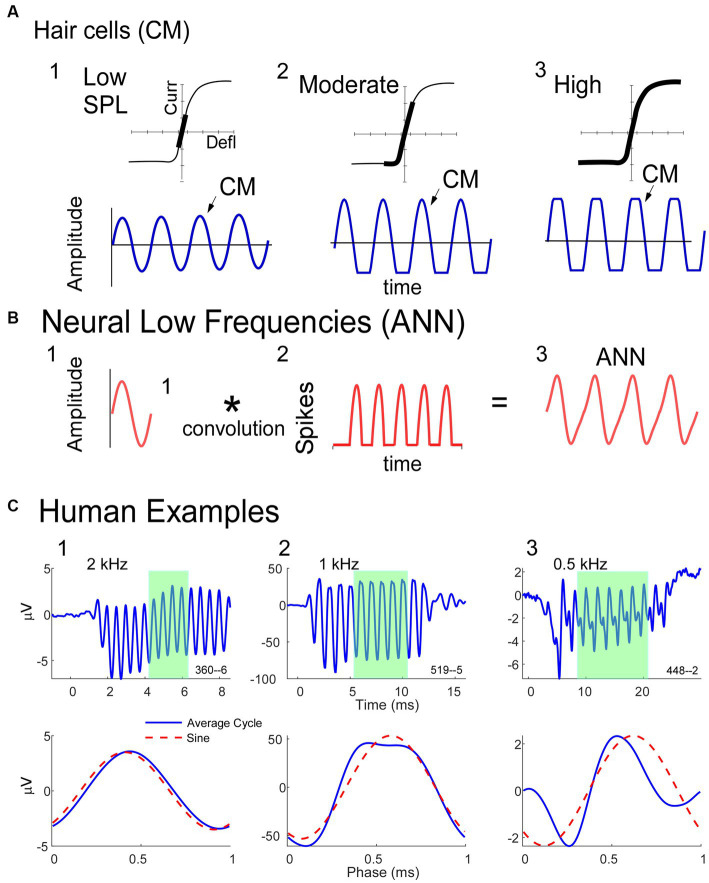
Schematic model of some of the biophysics of hair cell and neural sources for the ECochG potentials. **(A)** The CM is produced by the opening and closing of channels in the stereocilia of hair cells. To low intensities (left) the response is within the linear part of the operating range (top) and the CM produced is sinusoidal at the stimulus frequency (bottom). With increased intensity there will be asymmetric saturation (middle) to the degree the operating point is offset from the middle. To high intensities symmetric saturation will occur (right). **(B)** The ANN is produced by the phase-locked firing of auditory nerve fibers to low frequency sounds (<~1.5 kHz in gerbils and humans). It can be modeled as the convolution of a unit potential, or shape of the action potential as it appears at the RW, with the distribution of action potentials across all fibers that produce the ensemble response. **(C)** ECochG to 90 dB nHL tone bursts from three human CI subjects. The top row is the time waveform to condensation phase and the bottom is the ‘average cycle,’ or average of the cycles during the steady-state response (from shaded regions in top row). 1. In this case the average cycle shows little distortion to a 4 kHz tone burst, consistent with its being above the phase-locking range for the ANN and in the linear region of the CM (as in **A**, left). 2. In this case the response to a 1 kHz tone burst shows asymmetric saturation (as in **A**, middle). 3. Responses to a low frequency (0.5 kHz) tone burst. Here there is extensive distortion in the average cycle but not of a type that can be produced by hair cells, so is due instead to the ANN.

A description of some of the biophysical elements that produce the CM and ANN are shown in Figure 1. The CM (Figure 1A) is produced by currents flowing through stereocilia as mechanosensitive transduction channels open and close with basilar membrane movement. When the sound level is low, the stereocilia are in the linear part of their operating range (Figure 1A1) and the CM produced to a tonal input (bottom row) is essentially sinusoidal. As the sound level increases (Figure 1A2), output saturation is reached, but not symmetrically. Typically, more channels are closed than open at rest, producing saturation first in the hyperpolarizing direction of stereociliary movement. The asymmetry in the saturation is greater for inner than for outer hair cells (40). At high levels (Figure 1A3) saturation is to both directions, but the asymmetry remains. Thus, the shapes of the ECochG response produced by the CM will either be sinusoidal (Figure 1A1), asymmetrically saturated (Figure 1A2), or with a symmetrically saturated component as well (Figure 1A3).

The ANN is produced by the summed, synchronous activity of nerve action potentials as they phase-lock to the stimulus fine structure. Based on the extensive literature describing the CAP, which is the summed, synchronous activity of auditory nerve fibers to stimulus onsets (41–44), the ANN (Figure 1B) can be described as a unit potential (Figure 1B1), or shape of an action potential as it appears at the RW, convolved with the distribution of action potentials coming in a cyclic fashion from individual auditory nerve fibers (Figure 1B2). Because the spike rate cannot go below zero, the auditory nerve output is a rectified version of the stimulus input. The shape of the unit potential resembles an action potential with positive and negative components, so when convolved with the PST histogram the ANN (Figure 1B3) has positive and negative components.

Examples of how ECochG responses follow these principles in humans CI subjects are shown in Figure 1C. For each of three cases, the top row shows the ECochG time waveform to condensation phase stimulation, and the bottom row shows an “average cycle,” or average of each cycle in the steady-state response (shaded regions in top row). The average cycle is equivalent to a period histogram, a common representation of the cycle PST in Figure 1B2. It will be our primary unit of analysis for this report. For case 1, there was no ANN since the frequency (4 kHz) was above the phase-locking range, and the average cycle was sinusoidal (as in Figure 1A1). For Case 2, the average cycle (to 1 kHz) had a shape characteristic of asymmetrical saturation, as in Figure 1A2. Case 3 (right) shows the response to a low frequency (0.5 kHz) tone burst. Here, the average cycle does not match one of the possible shapes for the CM (Figure 1A), so the presence of an ANN is indicated.

#### The average cycle as the unit of analysis for detecting and measuring the CM and ANN

As we will describe in the next sections, we have developed methods for detecting the presence of the ANN and estimating its magnitude, along with that of the CM, using the average cycles as the main unit of analysis. Previous methods to identify the ANN have been primarily spectral (46–48) or used masking (49–52). Spectral methods rely on the 2nd harmonic in the summed responses to the two phases, under the assumption that the rectified responses to opposite stimulus phases will interleave to form what has recently been called the ‘Auditory Nerve Overlapping Waveform” (48, 53). At low and moderate intensities, the second harmonic is predominantly neural, so the ANOW is expected to be proportional to the ANN. However, the ANN is periodic with the stimulus frequency, so most of its energy is in the 1st harmonic, where it overlaps spectrally with the CM. In addition, at moderate and high intensities some of the second harmonic can be from asymmetric saturation of the CM, so the size of the second harmonic does not provide a quantitative estimate of the ANN. The other approach is masking, under the assumption that neural responses will adapt while HC responses will not. Masking can demonstrate the presence of the ANN but the proportion that is masked is dependent on the time and frequency relationships between masker and probe. In addition, obtaining a reliable data set using masking is not feasible while monitoring ECochG during a CI surgery or clinic visit.

The approach we have used is to estimate the CM and ANN using a model where the average cycle is the input and then equations developed from Figure 1 are used to estimate the amounts of CM and ANN that produce the best fit (45). Here we are augmenting this model with a deep learning algorithm (DLA) to first identify cases with or without ANN. The purpose of the DLA is to correct an issue with the analytic model, which estimates at least a small ANN even to high frequencies above the phase-locking range. This result is because the CM-only responses can deviate slightly from the expected shapes (45). The DLA makes no assumptions about expected shapes.

### Acoustic stimulation and recording

The acoustic stimulation and recordings of cochlear responses in both gerbils and humans were performed using a Bio-logic Navigator Pro (Natus Medical Inc., San Carlos, CA) as described previously (29, 45, 54, 55). The speaker was an Etymotic, ER-3B. The recording electrode was a stainless-steel probe of the type used for facial nerve monitoring during CI surgeries (Neurosign 3,602-00-TE, Magstim Co., Wales, UK), placed at the round window in both gerbil and humans. For human subjects, surface electrodes over the contralateral mastoid and on the forehead, and for gerbils, needle electrodes in the neck muscles and tail, served as the inverting and reference electrode, respectively. Gain was 1,000x for gerbils and 50,000x for humans. In some cases, for both gerbils and humans the sound tube was crimped which removed the responses, indicating that they were not contaminated by electrical artifact.

Stimuli were tone bursts alternating in condensation and rarefaction phases, with 100 (gerbil) or 250 (human) repetitions to each phase. Tone burst frequencies were 250, 500, 750, 1,000, 2000 and 4,000 Hz. Calibration was performed using a ¼” microphone and measuring amplifier (Bruel & Kjaer, Naerum, Denmark) and a 2 cm brass chamber for humans, and using a probe tube in the closed field in the ear canal of gerbils. High-pass filter settings for the recordings was 10 Hz in humans and 1 Hz in gerbils, and low-pass settings were 5,000 Hz (250–1,000 Hz tone frequencies), 10,000 Hz (for 2000 Hz) or 15,000 Hz (for 4,000 Hz tone frequency).

### Gerbil and human data sets

We present ECochG data from RW recordings in gerbil and human subjects with different hearing conditions (Table 1). For gerbils entering the recording part of the study, the auditory status was either normal-hearing (NH) or high-frequency-noise-induced hearing loss (HF-NIHL). Animals were classified as NH if untreated by noise or pharmacological agents prior to the experiment, and if ECochG signal magnitudes and thresholds were within the “normal” range for NH animals observed in this and previous experiments (45, 56). At 4 kHz, for example, CM magnitudes to 90 dB SPL were > 40 dB SPL and thresholds <10 dB SPL in all animals. In the HF-NIHL animals, the cut-off frequency of the 122 dB SPL noise was 4 kHz, which corresponds to approximately halfway along the characteristic frequency regions of the gerbil cochlea (57). In previous studies we showed that both outer and inner hair cells were removed in basal parts of the cochlea in response to the intense noise exposure used (58, 59). For both types of hair cells the transition from few or no hair cells to complete preservation was sharp and showed little variability as a percentage of distance from the apex of the basilar membrane compared to the total length, across animals (OHCs = 49.8 ± 4.50% and IHCs = 58.9 ± 4.46%, *n* = 19, errors are standard deviation). The more basal transition zone for IHCs showed a greater resistance to the noise, and there were often some IHCs preserved in the hook region of the cochlea as well. A few cases in the previous studies showed less or no hair cell loss, and, similarly, some cases here had CM thresholds that overlapped with normal hearing animals and were excluded. All of the HF-NIHL animals used here had CM thresholds to 4 kHz and higher that were > 50 dB SPL compared to an average of 0 dB SPL for the NH animals. The HF-NIHL condition was intended to mimic that of CI many subjects, where residual hearing, if present, is typically restricted to the apical half of the cochlea (frequencies <1.5 kHz in humans, <4 kHz in gerbils). Both gerbil groups (NH and HF-NIL) were also studied after the neurotoxin kainic acid (KA) was applied to produce cochlear synaptopathy. The KA was 60 mM in artificial perilymph consisting of (in mM): 127.5 NaCl, 3.5 KCl, 25 NaHCO3, 1.3 CaCl2, 1.2 MgCl2, 0.75 NaH2PO4, and 11 glucose, and pH adjusted to 7.3 with HCl (60), heated to 37 degrees C. It was placed at the RW for 1 h. With this protocol the loss of auditory nerve response is nearly total for the basal cochlea, but less so for the apical, due to the cochlea’s diffusion characteristics [see an anatomical image of the effects of KA in Figure 4 of Pappa et al. (56)]. Some of the NH animals here are the same as from the Pappa et al. study, and the same criterion for inclusion after KA was used. This criterion was that an increase in CM threshold between pre and post KA responses to 4 kHz, which is above the phase-locking range where the ongoing response is purely CM, i.e., from hair cells and thus not expected to be affected by the KA, had to be within 3 dB. This criterion resulted in exclusion of three animals.

**Table 1:** Data sets.

Species	Hearing condition	Cases
Gerbil	NH^1^	54
Gerbil	NH (post KA)^1^	20
Gerbil	HF-NIHL^2^	10
Gerbil	HF-NIHL (post KA)^2^	7
Human	CI^3^	166
Human	Non-CI^3^	42

^1^Normal hearing. ^2^High-frequency noise induced hearing loss. ^3^Cochlear implant.

The human subjects comprised surgical patients where the RW was accessible intraoperatively and included both CI and non-CI subjects. The CI subjects spanned all age groups. The non-CI subjects were undergoing surgery for a vestibular schwannoma or for Ménière’s disease, except for one subject who had a tumor removed from the jugular foramen allowing access to the RW.

### Deep learning algorithm

**Figure 2:**
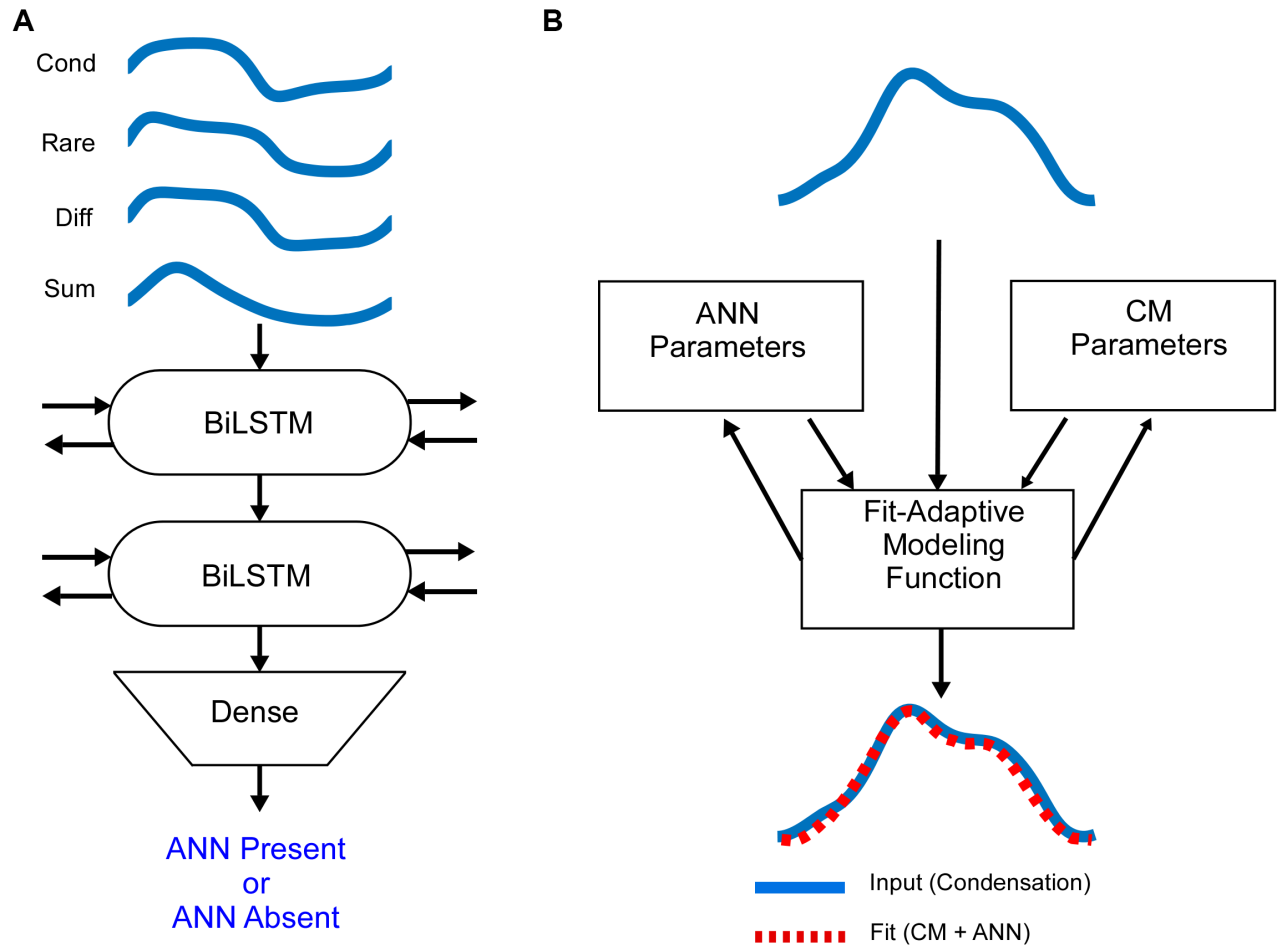
Mathematical models **(A)**. The average cycle for condensation, rarefaction, difference and alternating curves, normalized to amplitude and shifted to start at zero phase, were used as the input to the DLA. The network consisted of two layers of Bidirectional Long Short Term Memory (BiLSTM) nodes, followed by a dense node (see text and Material and Methods for further descriptions). The output was either ANN-present or ANN-absent. The training data was from NH animals, where low frequencies are ANN-present and high frequencies are ANN-absent. **(B)** Schematic of the biophysically based model used to determine the amount of CM and ANN. The input to the model is the average cycle to condensation phase stimuli. It is then modeled by a fit-adaptive function which updates ANN and CM parameters (see text) to produce an output which best matches the input [from (45)].

Since responses to low frequencies in an NH animal should always contain an ANN, while those to high frequency should not, these represent an ideal training set for a DLA to recognize its presence or absence in an average cycle. The input to the DLA (Figure 2A) was a tensor of the average cycles defined by condensation, rarefaction, difference, and alternating waveforms, using the responses to phase-alternating stimuli. These average cycles were normalized for amplitude and for 0 starting phase. The DLA was an implementation of Bidirectional Long Short-Term Memory (BiLSTM) layers, which are a specific subtype of recurrent neural networks that are frequently used on sequence data because they have an increased memory for events that are distant from each other (61). The bidirectional nature of the BiLSTM provides information about dependencies from both the forward and backward direction at every point. Finally, a dense layer with a sigmoid activation function was used to encode each of the ECochGs as either ‘ANN present’ or ‘ANN absent.’

The DLA was implemented in Python using the Keras library with TensorFlow as a backend. The architecture of the network was an input layer, followed by two BiLSTM layers with 50 recurrent units each, and finally a dense layer with a sigmoid activation function. The DLA was compiled with parameters to search for the best accuracy using a binary-crossentropy loss function with the adam optimizer.

The DLA weights were trained on the training set for 500 epochs, after which the increase in epoch accuracy and epoch loss leveled off and were validated on the test set. The final weights were saved so that they could be loaded and used for future classification tasks.

Because only the shapes of the cycles, and not amplitude or phase, are considered, the results from the neural network are generalizable for different experimental data sets. Each of the gerbil and human data sets were collected with the same recording and stimulation equipment and parameters.

### Analytic model and ANN proportion

Once an ECochG is labeled as ANN Present or Absent, we use a fit-adaptive function to model the contributions of the ANN and CM based on a depiction of the biophysical properties that produce each in Figure 1, as shown in Figure 2B (45). Briefly, the model mathematically describes the shapes of the CM and ANN and convolves them to create an average cycle of the ECochG, which it compares to the known signal and adjusts parameters until the error is minimized. Differences from the previous study were that the average cycles were interpolated to 100 points using a spline instead of linear interpolation, and several different starting parameters were run to help avoid local minima. Parameters for the CM were amplitude, phase, and differential saturation of the peak and trough, and for the ANN were amplitude and a ‘spread of excitation’ parameter that allowed the cycle histogram to increase in width to account for summation across fibers with varying phase. The model optimized these parameters and reported the values for the CM and ANN that produced the best fit. From these values, the proportion of ANN was simply $ANN\;\text{proportion}=\frac{ANN}{CM+ANN}$, where CM and ANN are their respective amplitudes in μV.

## Results

### The complex shape of average cycle is caused by the ANN

**Figure 3:**
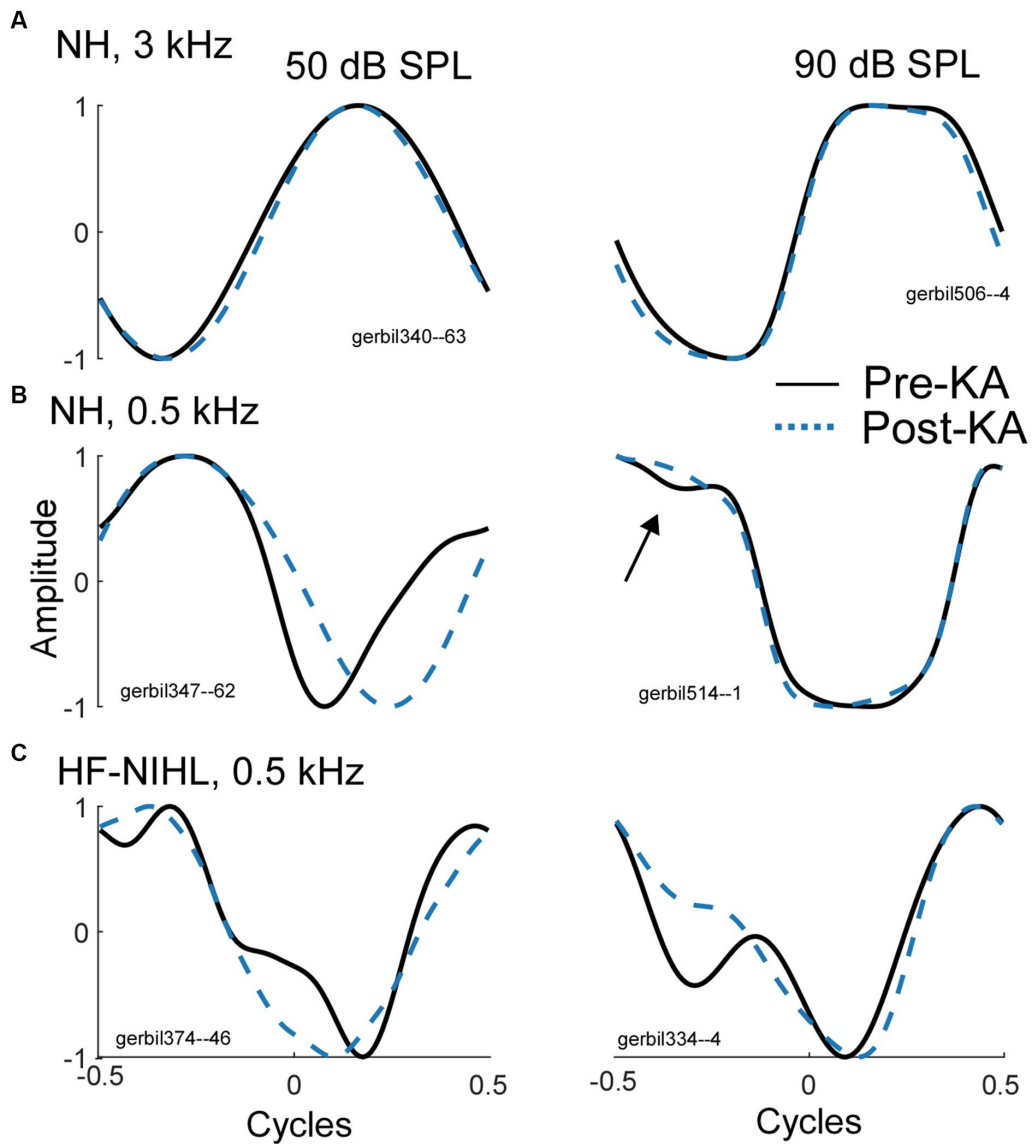
Effects of the neurotoxin KA to produce cochlear synaptopathy. **(A)** After application of KA, the responses to a high frequency (3 kHz), whether sinusoidal (left) or saturated (right) did not change, indicating no ANN. **(B)** To low frequencies (500 Hz) in NH animals the curves became simpler and consistent with that expected for CM-only. **(C)** To low frequencies from HF-NIHL animals also simplified after the KA, but pre-KA were even more distorted than in the NH cases, and the effect of KA appeared to be only partial, as was also common to low frequencies in some cases for NH animals.

In gerbils, it can be demonstrated that a complex shape of an average cycle, not expected from the CM alone (Figure 1) is caused by the ANN. In Figure 3, examples are shown before and after KA was applied to the round window. To a high-frequency tone burst in an NH animal (Figure 3A, 3 kHz), the average cycle had a sinusoidal shape to a moderate intensity (50 dB SPL, left), while to a high intensity (90 dB, right) it was asymmetrically saturated. Both shapes are characteristic of the CM-only waveform, and the KA had little effect. In contrast, to a low-frequency stimulus (Figure 3B), the shape prior to the KA was not consistent with a CM-only response, while after KA the average cycle was sinusoidal to the moderate intensity and was saturated to the high intensity. The effect of KA at the high intensity was subtle (arrow), but the small deviation in the pre-KA shape was a consistent feature not seen in cases that did not have an ANN. In Figure 3C there is a further example of a low-frequency response, but this time in an HF-NIHL animal. Here, two features stand out: the pre-KA curves are particularly distorted to both low and high intensities, and the effect of the neurotoxin appears to be incomplete. The high degree of pre-KA distortion for HF-NIHL animals compared to NH animals was a characteristic result further considered below. A partial effect of the KA was common to both gerbil groups and is presumably because of incomplete diffusion to the apex, so some ANN remains. This effect is minimized in NH animals at high sound levels, where the largest part of the response is from the base of the cochlea where the removal of the neural elements with KA is more complete.

### Performance of the DLA model

The model was trained and tested on 1764 average cycles from the 54 NH animals, including 641 to low frequencies (0.25–0.75 kHz) and 1,123 to high frequencies (2–6 kHz). The expectation is that in NH animals the responses to low frequencies within the phase-locking range (<2000 Hz) will all contain an ANN, while none of the frequencies above that range will. Thus, these represent “true known” responses.

**Figure 4:**
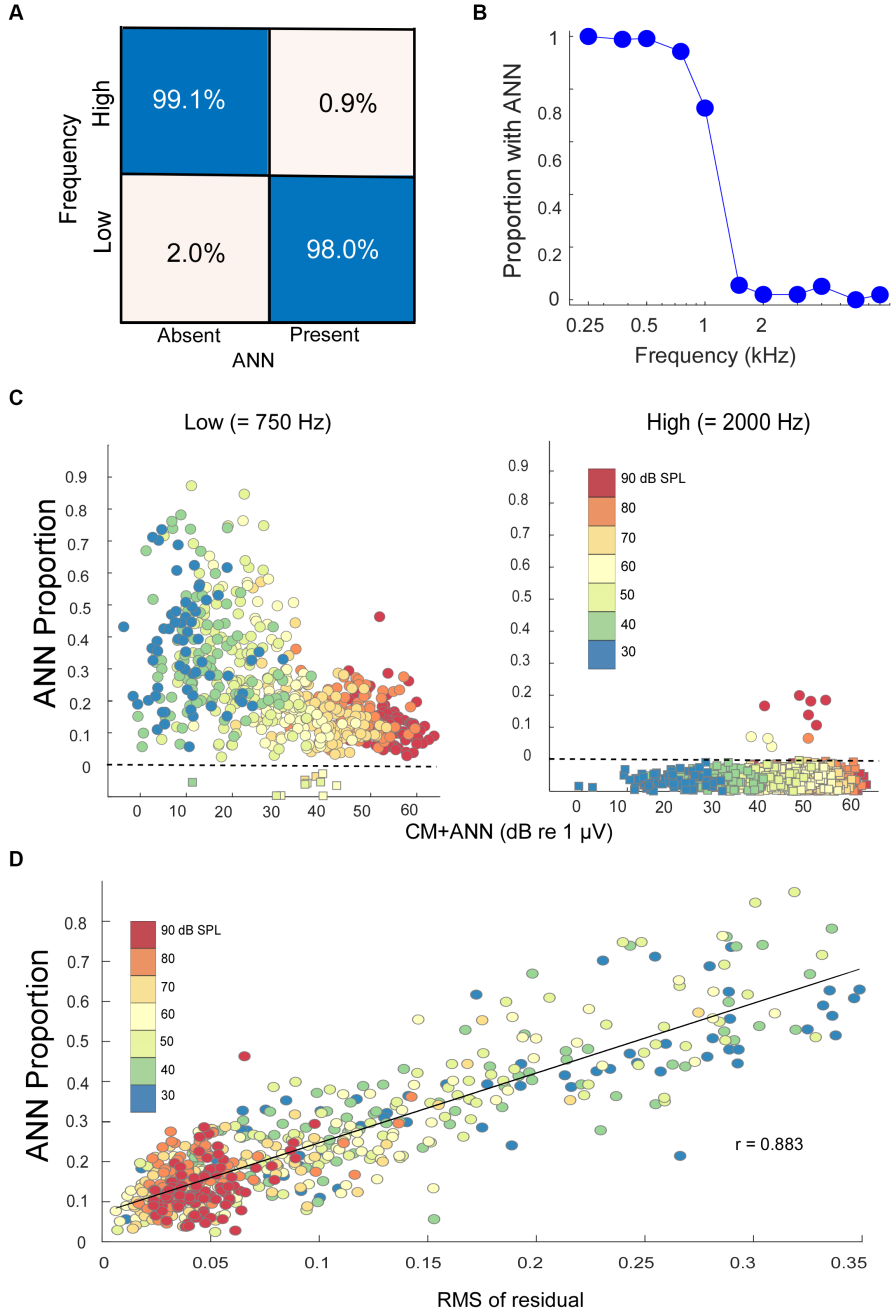
Modeling results. **(A)** Confusion matrix of the results with NH animals, confirmed with an independent test set, indicating the DLA was able to correctly identify and reject the presence of ANN at a high rate. **(B)** Percentage of the cases where an ANN was detected from the DLA as a function of stimulus frequency in NH animals. **(C)** The ANN proportion as a function of the size of the CM + ANN, both reported by the model, for NH gerbils to low and high frequencies. For cases with no ANN according to the DLA, the model was run without the ANN component. Each symbol is the ANN proportion to a frequency/intensity combination, so there are many points for each gerbil. The square symbols below the dotted line had no ANN according to the DLA, and were plotted at 0 with jitter added to make the points more visible. **(D)** Comparison of the ANN proportion with an independent method of estimating relative size of the ANN. The metric is the root mean square value of residuals in a regression of each low frequency average cycle with its best fit among the high frequency cases. The high correlation (*r* = 0.883) indicates the CM and ANN are reported from the analytic model in reasonable proportions.

The data was split 70/30 into training and test sets. The model weights were trained for 500 epochs, after which the increase in epoch accuracy and epoch loss leveled off. With this distribution, the model had a sensitivity for correct detection of an ANN of 99.1%, and a specificity for correct rejection of an ANN of 98.0% (Figure 4A).

When considered on a frequency-by-frequency basis, the proportion of average cycles identified by the DLA as having ANN was >95% for 0.25 to 0.75 kHz, which then dropped to <5% for frequencies of 1.5 kHz and higher (Figure 4B). The proportion at 1 kHz was about 75%. The overall phase-locking to 1 kHz may be reduced because it is near the cut-off frequency for both gerbils and humans, but a reduction in the ANN may also be because the width of a unit potential begins to exceed the width of a cycle of phase-locking (47). Consequently, 1 kHz was not used in the analyses of ANN proportion.

### Analytical model results

In the NH animals, to low-frequency tones (250, 500, and 750 Hz), the ANN proportion decreased as the CM increased (Figure 4C, left). The color scale shows this relative increase in the CM compared to the ANN to be largely a function of intensity. The relatively few that were excluded by the DLA are shown below the line rather than at zero with some jitter added for clarity. To high-frequency tone bursts (≥2000 Hz), most average cycles were reported by the DLA to have no ANN (Figure 4C, right).

There are more parameters than equations in the analytic model, so the fits do not necessarily represent unique solutions to the parameters. We used multiple starting points to avoid local minima but fits at some distance from the correct values can occur from parameter optimization. A check that the model was estimating reasonable values was to compare its output to that of an independent method of analyzing an average cycle which did not make assumptions about the shapes that make up the CM and ANN. For each low-frequency average cycle, we performed a cross-correlation with all the average cycles to high frequencies and measured the root-mean square value of the residuals in the case with the closest fit. The idea was that the smaller the residuals, or the closer a match to a CM-only case, the less ANN would be present. We found that this metric correlated well (*r* = 0.883) with the ANN proportion (Figure 4D), suggesting that the analytic model, which provides values the ANN and CM, scales with actual rather than far distant values.

### Effects of neurotoxins and noise exposure on the ANN proportion in gerbils

**Figure 5:**
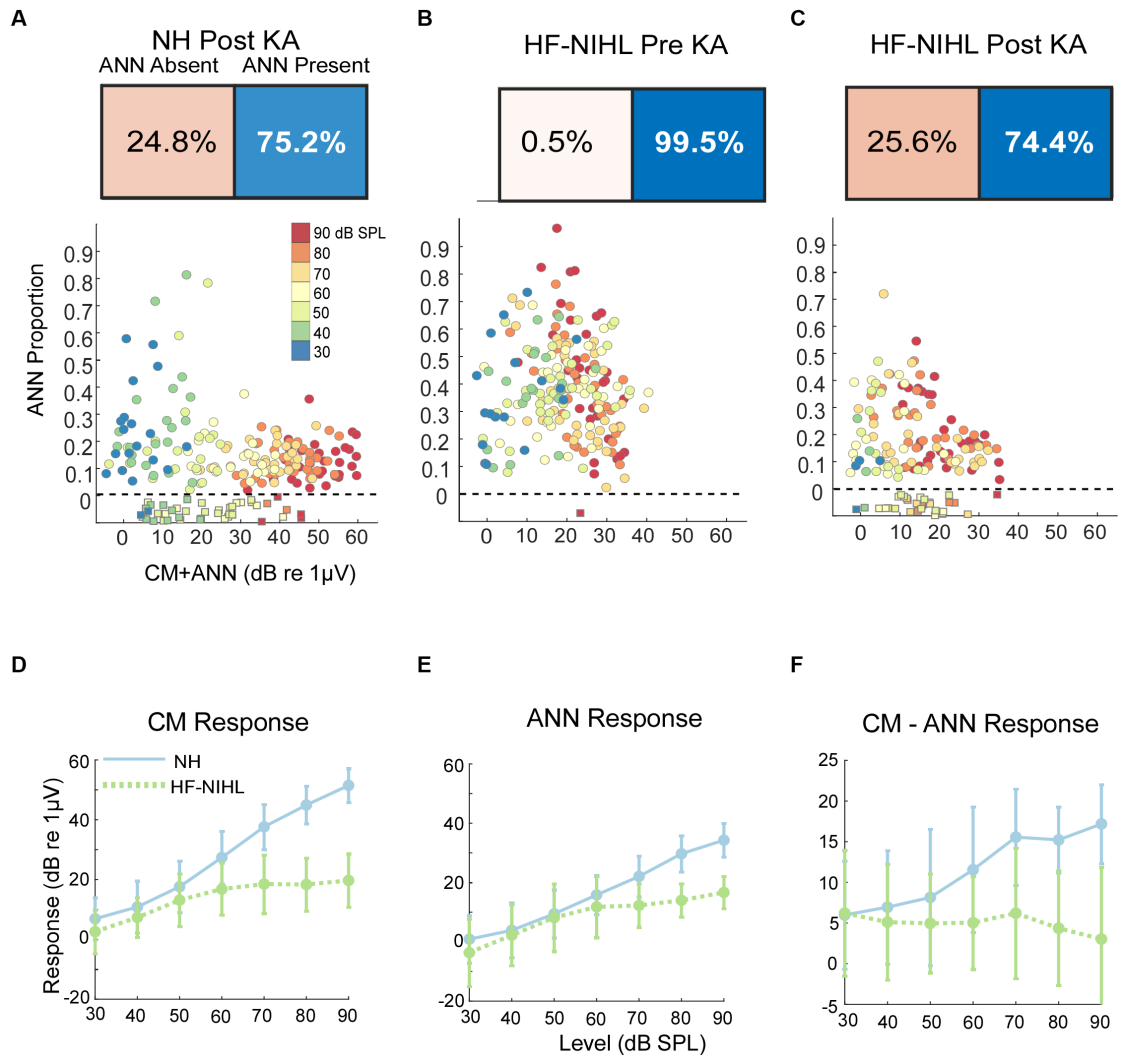
Results in gerbils. **(A)** Effects of the neurotoxin KA on low-frequency average cycles. There is a higher proportion of ANN-absent cases and less ANN overall than prior to KA (compare with **B**). **(B)** Results to low frequencies from HF-NIHL cases. The CM is reduced, but the proportion of ANN is high. **(C)** Results from HF-NIHL cases after KA. There is an increase in ANN-absent cases and in the ANN proportion of most cases. **(D)** Comparison of the CM in NH and HF-NIHL animals (pre-KA). The CM saturates in the HF-NIHL animals, due to limited spread of excitation because the basal cochlea is non-functional. **(E)** Comparison of the ANN. The effect in the HF-NIHL animals is similar but smaller than with the CM. **(F)** Difference between the CM and ANN. This difference grew in the NH animals due to spread of excitation but not in the HF-NIHL animals, where it was blocked. Error bars represent standard deviations.

As would be expected, post-KA, NH animals (Figure 5A) had a large increase in the numbers of responses to low frequency (<1,000 Hz) judged by the DLA to have no ANN (24.8% compared to 2.0% in NH animals from Figure 5A). Similarly, the ANN proportion was in general smaller, indicating less ANN relative to the CM than before the KA (mean = 0.33 ± 0.176 for the Pre-KA vs. 0.21 ± 0.13 for the Post-KA). However, since most cases still had some ANN the action of the neurotoxin was often only partial. Finally, the effect of intensity was decreased, with most cases showing a small ANN proportion even to low intensities. A quantification of this decrease in the effect of intensity is the slope of the best fit regression line through all of the data, which was 0.52/dB for the Pre-KA NH condition and 0.27/dB for the Post-KA condition.

For the HF-NIHL animals, prior to the KA the DLA again showed that almost all responses to low frequencies had an ANN (Figure 5B). The removal of hair cells from the basal half of the cochlea did reduce the CM, as expected. However, the HF-NIHL animals showed an *increased* proportion of ANN, especially to higher intensities, compared to the NH distributions in Figure 5C, which will later be better quantified. After KA (Figure 5C) the DLA again found more responses without an ANN, and the ANN proportion decreased, indicating partial to complete synaptopathy in most cases.

The growth of the CM (as computed from the model) is linear over the intensity range of 30–90 dB SPL, while for the HF-NIHL animals it saturates at a moderate intensity. The early saturation in HF-NIHL animals (Figure 5D) is because the spread of excitation toward the base of the cochlea as the intensity is raised is limited by the loss of hair cells. In contrast, the ANN (Figure 5E) in the NH animals grows at a comparatively slower rate, and the difference between NH and HF-NIHL animals is much smaller. The result is that the difference between the CM and ANN (Figure 5F) grows with intensity in the NH animals but does not in the HF-NIHL animals.

### Results in humans

**Figure 6:**
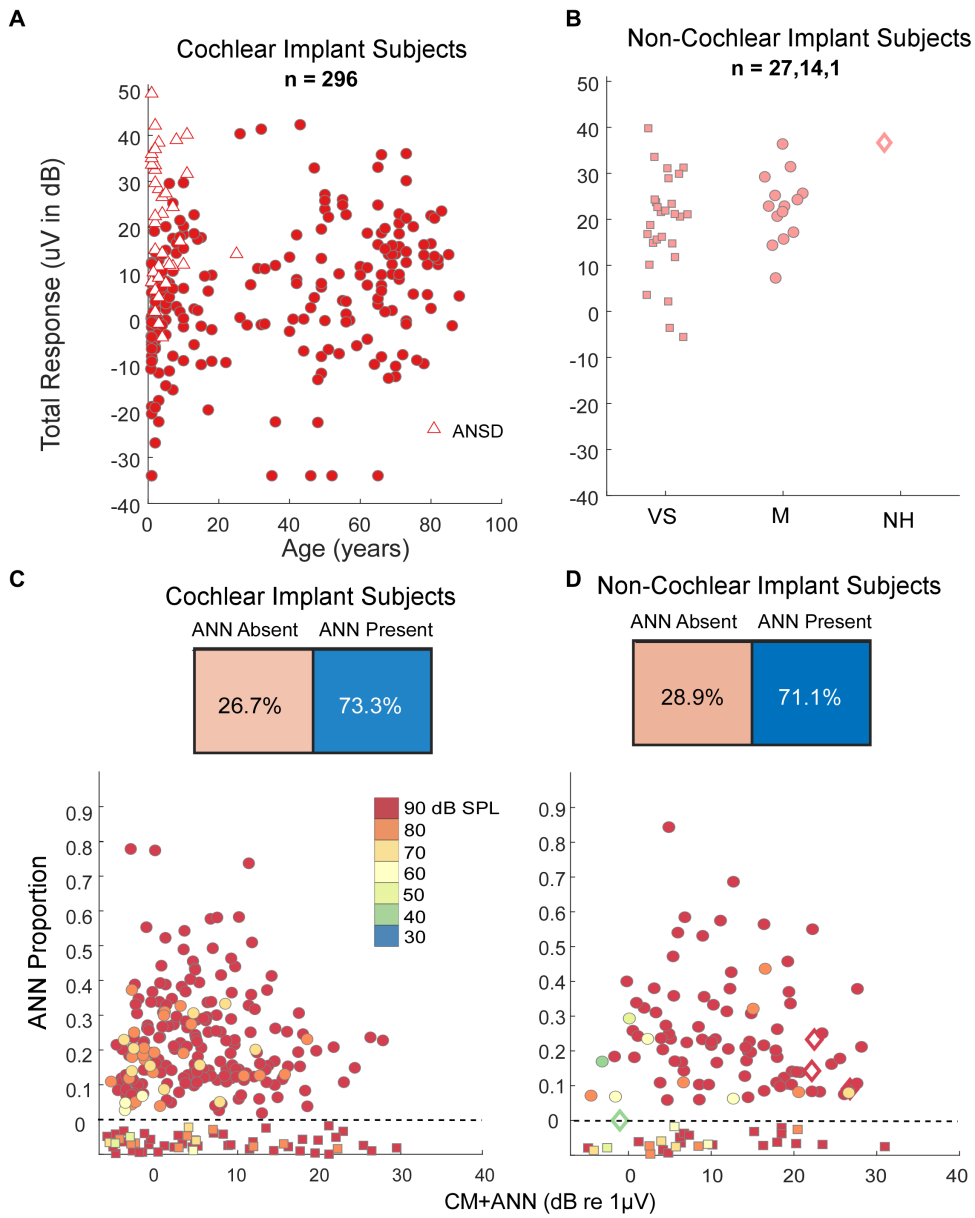
Results in human subjects. **(A)** Distribution of ‘Total Response’ (TR, a measure of the responses summed across frequencies, see text) in CI subjects. There is a wide range of responses independent of age, with the largest TR often seen in children with auditory neuropathy spectrum disorder (ANSD). **(B)** TR in non-CI subjects as a function of age. The largest TR in each group, including the case with no auditory-related syndrome and thresholds in the normal range, were similar to the largest in CI subjects. VS, vestibular schwannoma. M, Meniere’s, NH, Normal Hearing. **(C)** Output of the DLA and distribution of ANN proportion in CI subjects. There were many cases judged by the DLA to have no ANN, but otherwise there was a wide range of ANN present. **(D)** Output of the DLA and distribution of ANN proportion in non-CI subjects. Similar to the CI subjects, there were many cases judged by the DLA to have no ANN, but otherwise there was a wide range of ANN present.

Our human data sets were CI subjects and others where the round window was available during surgery. A metric used to characterize the overall responses from each subject is the ‘Total Response (TR),’ which is a summed measure of the output of the cochlea to a range of frequencies (29, 31, 62, 63) (see Materials and Methods). Specifically, the TR is calculated from the sum of the response magnitudes to each frequency, with the response at each frequency measured as the sums of significant peaks to the stimulus frequency and 2^nd^ and 3^rd^ first harmonics. The frequencies used were 0.25, 0.5, 0.75, 1, 2, and 4 kHz. The TRs for the CI subjects (Figure 6A) covered a wide range independent of age. For the youngest children many of the cases with large TR were auditory neuropathy spectrum disorder (ANSD) subjects, which is a condition characterized by loss or desynchrony of auditory nerve firing with preservation of cochlear function, and so may be related to cochlear synaptopathy. The TRs for non-CI subjects (Figure 6B), were on average larger than for the CI subjects, but interestingly the cases with the highest values were similar for each data set. Even the one subject with audiometric thresholds within the normal range had a level only near the maximum of the other groups, not above them.

To low frequencies (0.25, 0.5, and 0.75 kHz), the DLA reported that CI and non-CI (Figures 6C,D) subjects both had a large fraction (28.9 and 26.7%, respectively) with no detectable ANN. Of the remainder there was a wide distribution, including some with evidence of a strong ANN (e.g., >25% of the combined responses).

### Human groups are most similar to gerbil groups exposed to KA

**Figure 7:**
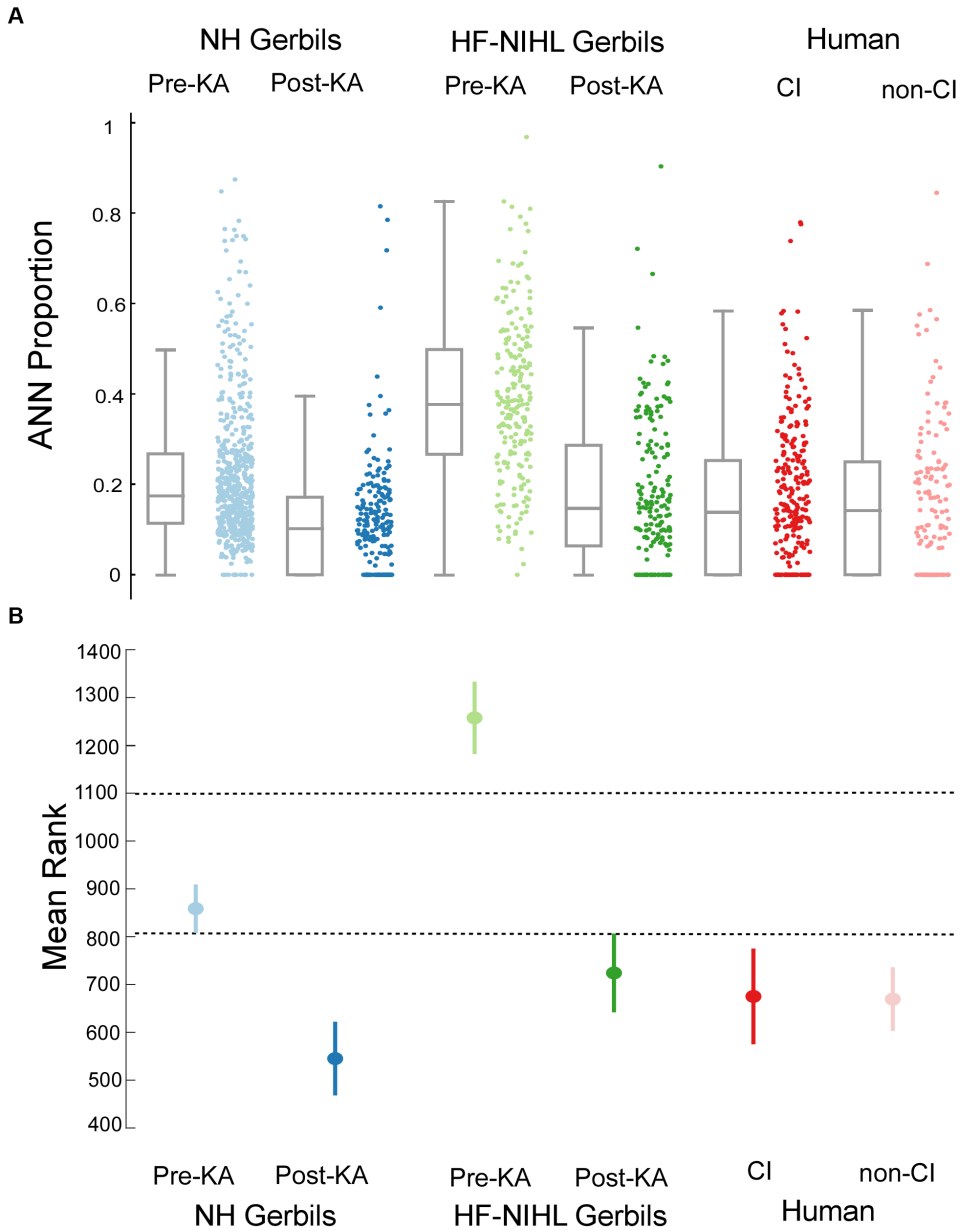
Comparisons across the gerbil and human data sets. **(A)** Scatter plots show the distributions of ANN proportion, and the box plots show median, semi-interquartile ranges, and whiskers that include 1.5 times the inter-quartile range. **(B)** Means and standard errors with correction for multiple comparisons (alpha = 0.083).

A comparison of the distributions of ANN proportion across the six groups is shown in Figure 7A. These distributions encompass different frequencies and intensities for each animal and human subject. The results of multiple comparisons, based on standard errors corrected for multiple comparisons (Figure 7B, alpha = 0.083), were that the pre-KA, HF-NIHL gerbils showed the largest proportion of ANN and was distinct from the other data sets. The gerbils with the next highest proportion of ANN were the pre-KA NH animals, and the ANN proportion was significantly greater than for any of the remaining groups. The distributions in both of the post-KA animal groups were not significantly different from each other or from the two human groups. Recall that the noise exposure for the HF-NIHL animals was intended to mimic that of subjects, particularly CI subjects, with high frequency hearing loss. Thus, if hair cell loss was the main cause of hearing dysfunction leading to cochlear implantation, the human CI subjects should have most closely resembled the pre-KA, HF-NIHL animals. Instead, their distribution, and the distribution of non-CI subjects, was most like that of the gerbil groups after application of neurotoxin that produced a complete or partial synaptopathy, which implies synaptopathy is present in the human subjects as well.

### ECochG thresholds are often better than behavioral in CI subjects

**Figure 8:**
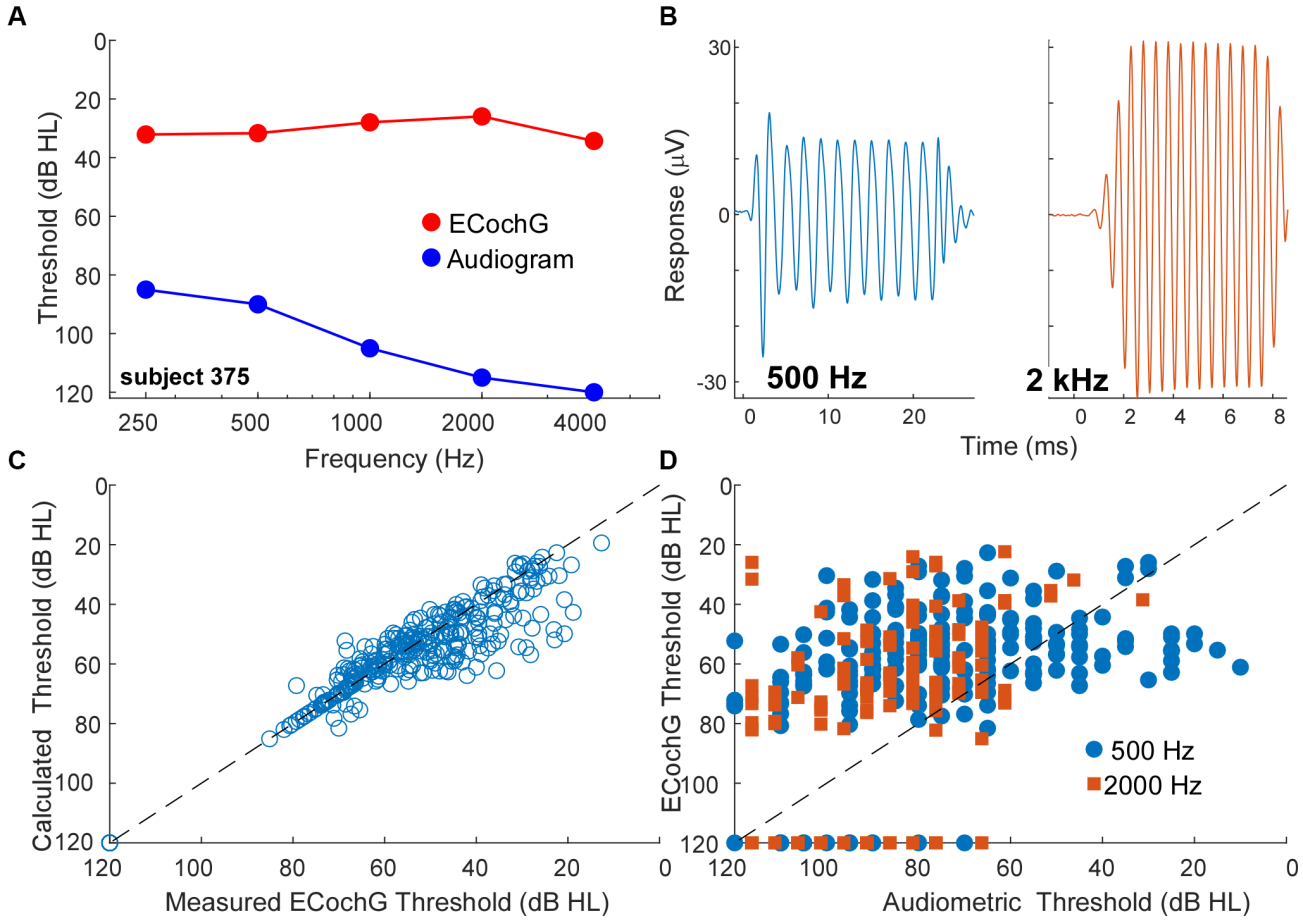
ECochG thresholds are more sensitive than behavioral thresholds. **(A)** An example of ECochG threshold vs. behavioral. Despite having severe to profound hearing loss as shown by the audiogram, the ECochG is only in the range of mild hearing loss. **(B)** ECochG responses to 500 and 2000 Hz at 90 dB nHL. **(C)** Calculated ECochG Thresholds perform better than Audiometric Thresholds for both 500 and 2000 Hz. **(D)** Calculated thresholds are mostly similar to measured thresholds. Any deviations were typically in the direction where actual thresholds were better than calculated thresholds.

**Figure 9:**
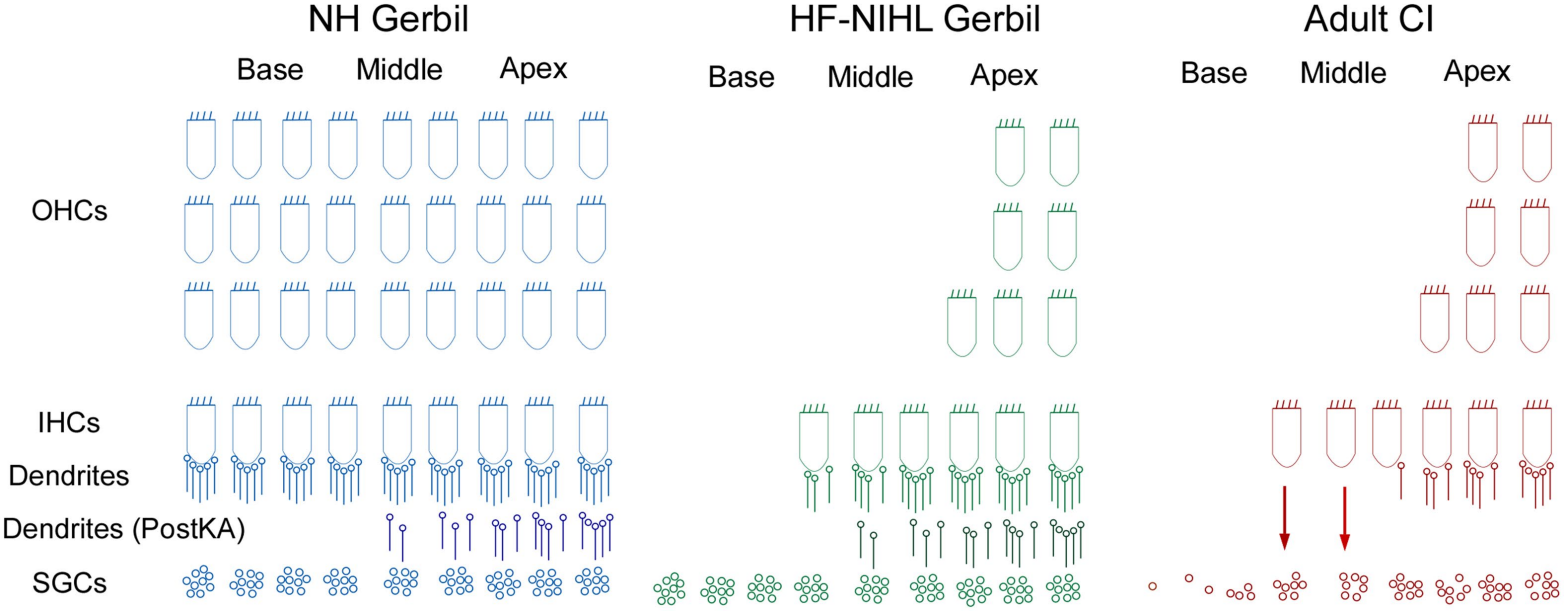
Schematic of the cochlear hearing postulated for each of the models used in this study. In NH gerbils, OHC, IHCs, and connections of ICs to spiral ganglion cells (SGCs) are present. In the Post KA condition, the dendrites between the IHCs are SGCs are severed in the basal cochlea but partially preserved in the apex. In HF-HIHL gerbils, the outer hair cells are removed to a greater extent than the inners, and in the Pre KA condition there is a normal complement of connected dendrites resulting in the increased overall proportion of ANN compared to NH animals. Post KA there is typically complete disconnection of dendrites from IHCs in the basal cochlea but only partial in the apical parts. The SGCs remain were the dendrites have been removed but are not visible in the recordings. In the adult CI subjects, the progressive hearing loss results in extensive hair cell loss in the basal cochlea, but at the transition zone to relatively preserved hearing there are groups of presumably primarily IHCs that are still present but with severed connections to the SGSs. The presence of hair cells and a functional organ of Corti may allow for continued production of trophic substances (arrows) that support SGC survival. The distribution of remaining neural connections with IHCs more closely resembled the Post KA than the Pre KA condition.

A behavioral threshold is typically based on activity in one or a few sensory receptors that result in only a few action potentials (64, 65). In contrast, generation of an evoked potential requires the summed, synchronous activity of numerous responding elements. Therefore, a behavioral threshold is expected to be more sensitive than an evoked potential. However, in CI subjects the ECochG threshold, recorded perioperatively, was often more sensitive than the pre-operative audiometric thresholds. An example is shown in Figures 8A,B, where the subject had severe hearing loss to low frequencies (250 and 500 Hz) and profound hearing loss to higher frequencies (Figure 8A, blue). However, the ECochG responses to both 500 Hz and 2 kHz were large (Figure 8B) such that the estimated threshold from the ECochG was only in the range of mild hearing loss to all frequencies (Figure 9, red). Time for data collection in the operating room is limited, so for frequencies other than 500 Hz we collected responses only to 90 dB nHL. To estimate threshold, we used a linear interpolation where a 1 dB reduction in stimulus level produced a 1 dB reduction in response, and threshold was taken as a response level of 0.02 μV (−34 dB re 1 μV), which is the threshold sensitivity for a response to achieve significance under good recording conditions (see Methods for significance criteria). When time permitted, we performed a level series to 500 Hz in 10 dB steps to better estimate actual thresholds, interpolated between the last significant response and the first non-significant response. When compared to the thresholds calculated from 90 dB nHL responses they were similar (Figure 8C) and the differences were typically in the direction where actual thresholds were better than calculated from the 90 dB nHL response (below the line of equivalence). This result was because large responses tended to be saturated, so that reductions in level did not produce corresponding drops in responses for the first 10 to 20 dB. Errors in this direction would cause the actual sensitivity of ECochG thresholds to be underestimated.

A trend for better evoked potential activity than behavioral was present overall, as shown for 500 and 2000 Hz in Figure 8D. Here, points above the line of equivalence indicate better ECochG than audiometric threshold. Considering all frequencies from 250 Hz to 4 kHz, a two-way ANOVA showed a main effect of frequency, in that higher frequencies showed less sensitivity for both behavioral and ECochG thresholds (*F* = 58.5, df = 4, *p* < 0.0001), and a main effect of measurement type, with ECochG showing significantly better sensitivity than behavior (*F* = 25.8, df = 1, *p* < 0.0001). The interaction between frequency and response type was significant (*F* = 4.8, df = 4, *p* = 0.0008), because the effect of frequency was smaller with ECochG, i.e., ECochG thresholds showed less of an increase to higher frequencies than did the audiometric thresholds.

## Discussion

Using data from normal-hearing gerbils as a training set, a DLA was used to identify waveforms that contained a neural component, the ANN, in ECochG responses to low-frequency sounds. In waveforms judged to have an ANN present, an analytic model estimated its proportion in the overall response. The models developed were then applied to ECochG data from both gerbil and human subjects. The gerbil groups included NH and HF-NIHL animals, with the HF-NIHL group intended to mimic the primarily low-frequency hearing condition of many CI subjects. Both gerbil groups were also tested after a neurotoxin was placed on the RW membrane to induce cochlear synaptopathy. The human subjects were surgical cases where the RW was accessible, which included CI subjects, subjects with Meniere’s disease undergoing a labyrinthectomy, and cases with a vestibular schwannoma that was being resected. The human groups had ANN proportions similar to those of gerbils after the neurotoxin, indicating the presence of cochlear synaptopathy in the human groups. In addition, thresholds to ECochG in the human subjects were generally more sensitive than those in the audiogram, a further indication that the recordings were from hair cells disconnected from auditory nerve fibers.

### The deep learning algorithm to identify the presence or absence of the ANN

The average cycles are sequential data, so a logical choice of neural network architecture was a Recurrent Neural Network (RNN), which is designed to utilize sequential information. Past information is stored in state vectors, which are considered at each point, so dependencies on past information can be used. Bidirectional RNNs consider dependencies on both past and future information (66). Note that the DLA created makes no assumptions about the underlying biophysics and, because of the choice of training set, did not require any expert curation of features. The training set was from NH gerbils where all responses to low frequency sounds contain an ANN, but none of the responses to high frequency sounds do, since they are above the phase-locking range detectable with ECochG (47, 55, 67). The input was average cycles, normalized in amplitude and starting phase, so that the algorithm operates only on variations in shape. By using responses across multiple frequencies and intensities within each range, the samples included a wide spectrum of waveform shapes that exemplify ANN-present or ANN-absent responses. The shapers were different enough that the DLA had both a high sensitivity and specificity (>95% for both).

### The analytic model to estimate the proportion of the ANN and CM

The analytic model provides a quantitative estimate of the sizes of the CM and ANN in a given response. Other methods, whether spectral, masking, or our correlation-fitting analysis (Figure 4D), are proportional to the size of the ANN but are not similarly quantitative. The good fit between the ANN proportion and the correlation-fitting analysis of ANN strength (Figure 4D) indicates that the parameters used are sufficiently constrained to a reasonable range to account for CM and ANN magnitudes. However, the model is over-determined, so that the solutions are not unique. We therefore consider the quantitative values to be reasonably accurate in the aggregate but with a range of uncertainty in particular cases.

The theoretical basis of the model (Figure 1) does not include all the relevant parameters that can lead to complex shapes of the average cycle. In particular, additional modeling (not shown here) indicates that complex shapes for the CM can be created if responses from different parts of the cochlea with different phases both have some amount of saturation. However, the experiments with neurotoxins suggest that the main source of complex average cycles is neural in the form of the ANN, because most complex average cycles resolve to those described in Figure 1A as typical for a predominance of CM over ANN. Furthermore, complex shapes with responses to high frequencies are rare. However, the current model includes only a subset of the biophysics underlying the shapes of average cycles and that future versions could include more parameters.

The model showed an *increase* in the proportion of ANN compared to CM in the HF-NIHL versus NH animals’ response to low frequencies. The intense noise used (122 dB for 4 h) produced an almost complete loss of OHCs and IHCs to frequencies above the cut-off of 4 kHz (58, 59). An explanation for the increase in proportional ANN may relate to the loss of spread of excitation to the basal cochlea, which in the normal hearing case will dominate the responses. That is, as the intensity increases the responses recruited from the basal cochlea occur in-phase due to the speed of the traveling wave through the basal cochlea (68, 69). In contrast, when the responses are coming from the apical cochlea some have different phases due to the slowing of the traveling wave near the characteristic frequency region. Thus, the CM response will grow more slowly with intensity due to interference. In contrast, the neural response will spread but the action potentials will not cancel as sine waves do. In addition to phase issues, the neural potential that produces the ANN is also likely to reach saturation prior to the CM. Above threshold, the rate at which low spontaneous rate fiber comes out of the relatively refractory period, which governs the overall rate of skipped cycles, is not dependent on intensity, so the rate saturates at a moderate intensity (70). Viewed another way, because of phase-locking the maximum rate that a fiber can contribute is dependent as much on the frequency as the intensity, because the maximum rate is less than one spike per cycle (except in the case of peak-splitting to the lowest frequencies).

### Cochlear synaptopathy in animal studies

Cochlear synaptopathy, using noise exposure and anatomical verification, has been identified in several species including mice, guinea pigs, and macaques (1, 4, 71). Its hallmark is loss of synapses and neural activity in the face of preserved hair cells and auditory thresholds. The ability to preserve auditory thresholds is due to selective preservation of low-threshold, high-spontaneous rate fibers (5, 6). In studies using CBA/CaJ mice, the loss of synapses was found to be irreversible (1), and the possibility of permanent loss of synapses combined with preserved audiometric thresholds led to concerns of significant “hidden hearing loss” in humans (72). Recently, it has been shown that in other strains of mice and in guinea pigs the loss of synapses is not permanent (23–25). However, these studies also show that in animals with some degree of permanent hearing loss, there is a continued synaptopathy, suggesting cochlear self-repair mechanisms may not be stable through a lifetime.

### Cochlear synaptopathy in human subjects

Human temporal bone studies show a greater preservation of hair cells than neural structures in aging subjects and those with greater hearing loss (8–10). To test for a physiological correlate, we compared gerbil and human subject groups based on RW recordings, where cochlear physiology can be explored with a high signal-to-noise ratio. The shapes of the average cycles were similar between gerbil and human subjects and reflect the same biophysical principles that underly each (e.g., Figures 1, 3). This similarity allows for comparisons between humans and experimentally manipulated gerbils. There are, of course, major differences between the species. One is the size of the response at the RW, with human responses about an order of magnitude smaller than gerbils (maximums of tens rather than hundreds of microvolts). Smaller responses in humans are likely to be due to the increased size of the cochlea, where responses at the RW must travel through larger spaces than in small animals. Response magnitudes with intracochlear recordings during CI insertion in humans, where the electrode can be close to the source generators, can reach hundreds of microvolts [e.g., (73–75)].

Interestingly, the largest responses were the same between the CI and non-CI subjects, despite the greater degree of hearing loss expected in CI subjects. The largest responses of both CI and non-CI subjects were similar to the single human case with normal hearing. Many of the CI cases with the largest responses were children with ANSD (Figure 6A), a condition related to cochlear synaptopathy where neural or inner hair cell dysfunction is present, but cochlear responses can be relatively normal (76, 77). In a previous study in children receiving CIs, ANSD cases could have a substantial ANN, similar to a control group of non-ANSD children receiving CIs (78). What was different was a very large CM and negative-polarity SP to high frequencies in ANSD compared to non-ANSD children, which was typically not accompanied by a CAP. The explanation for the large negative SP is the loss of neural and/or inner hair contributions to the SP which have a positive polarity (56, 79).

In addition to a distribution of ANN proportion comparable to animals treated with neurotoxins, another indication of cochlear synaptopathy is the overlapping thresholds between ECochG and behavior in CI subjects. In many cases the threshold for ECochG responses were lower (better) than the audiometric thresholds, which is not the expected direction for an evoked potential compared to behavior. Previous studies have also shown thresholds for ECochG to be better than for audiometry in some cases (80–82). An important issue is calibration, since in our study and most others the ECochG and audiometric thresholds are measured at different times and with different equipment. However, one study (82) measured ECochG through the implant in the clinic and audiometric thresholds at the same session with the same equipment, and this study also reported many ECochG thresholds to be better than behavioral. A better threshold for an evoked potential is not expected because a behavioral threshold can be obtained from very few active fibers (64, 65), while an evoked potential is determined by the summed, synchronous activity of many responding elements. Thus, hair cell function that is better than behavioral sensitivity is an indication of cochlear synaptopathy in CI subjects.

Finally, it has been shown in adult subjects and children implanted at greater than 6 years of age that larger ECochG magnitudes are associated with better speech perception outcomes with electrical hearing (29). These results contrast with preoperative tone audiometric thresholds, which are not predictive of speech perception outcomes with the implants (32, 34, 83). The explanation proposed is that some of the ECochG responses are from hair cells disconnected from auditory nerve fibers, i.e., cochlear synaptopathy, and that the presence or absence of functional hair cells indicates overall ‘cochlear health.’ This cochlear health is then indirectly related to the functional status of the auditory nerve available for electrical stimulation. Illustrations of the expected hearing conditions studied here are shown in Figure 9. In NH animals, all of the elements of OHC, IHCs, and connections of ICs to spiral ganglion cells (SGCs) are present. In the Post KA condition, the connections between the IHCs are SGCs are severed in the basal cochlea but partially preserved in the apex due to diffusion characteristics. In the HF-NIHL animals, the OHCs are removed to a greater extent than the inner (58), and in the Pre KA condition a nearly normal complement of dendrites exist to the remaining hair cells, resulting in the increased overall proportion of ANN compared to NH animals. As with NH animals, the KA then causes a similar variable but typically partial removal of the neural input. The SGCs remain viable for the 1-month interval between exposure and ECochG but are not visible to the ECochG. In the adult CI subjects, the progressive hearing loss results in extensive hair cell loss in the basal cochlea, but at the transition to relatively preserved hearing there are groups of primarily IHCs that are still present but with severed connections to the SGCs. These hair cells are visible to ECochG but not the audiogram. In addition to the hair cells themselves, the organ of Corti, endolymphatic potential, tectorial membrane and other features that support hair cell transduction must also be functional and can provide trophic substances (arrows) such as growth factors and neurotrophins that support SGC survival (84, 85). In the absence of this support, a greater proportion of SGCs are removed and the information provided by electrical stimulation is reduced. Although the hair cell distribution in the CI subjects and HF-NIHL animals are similar, the remaining connected neural portion more closely resembles the Post KA than the Pre KA condition.

There is currently a major effort underway to investigate the use of ECochG as a real-time monitor of cochlear health during CI surgery to detect and possibly lessen cochlear trauma during implantation and thereby improve hearing preservation and speech perception outcomes [reviewed by (86)]. The presence of cochlear synaptopathy in these subject effects the interpretation of the ECochG recordings. Current methods focus primarily on the responses to a single frequency, typically 500 Hz, delivered at high intensity (>100 dB SPL), to monitor changes from the apical electrode as the insertion progresses (75, 86–88). To a 500 Hz stimulus at high intensity, the recordings are primarily, although not exclusively, the CM, which because of cochlear synaptopathy will not be directly reflective of acoustic hearing. While changes in these responses can be a useful indicator of trauma that is worthwhile to avoid because it can lead to worsening of subsequent effects, such as foreign body response and fibrosis, such changes are unlikely to directly reflect the degree of hearing preservation to be expected. Thus, an additional fruitful focus as a monitor for trauma in hearing preservation cases could be the ANN, which is more directly related to neural preservation.

In cases where the RW recording was available during labyrinthectomy or during an acoustic tumor removal, the distribution of TR in these subjects was on average higher than for CI subjects, but the maxima were similar. These subjects often have a hearing loss due to endolymphatic hydrops, compression of the auditory nerve, or effects on blood supply to the cochlea (89–91). Still, the hearing loss is generally less than in CI subjects. However, like the CI subjects, the distribution of ANN-present and ANN-absent subjects was similar to gerbils with partial loss of synapses from neurotoxin applied to the RW, indicating a degree of cochlear synaptopathy in these subjects as well.

## Limitations and future directions

The human data revealed many cases where an ANN was not detected by the DLA, yet in some of these cases hearing, especially in non-CI subjects, was present. Consequently, it appears that an ANN can exist without detection by current ECochG. In these cases, basal hair cells may be present and dominate the responses, while surviving neural responses from the apex, or from within the core of the auditory nerve, are too small to be detected. This pattern would also be an indication of cochlear synaptopathy in the more basally located hair cells.

The potential benefit of a condition with functional, surviving hair cells is in the realm of neural regeneration. If regeneration is required to reintroduce hair cells and a functional organ of Corti, the prospect is daunting. However, if only a reconnection between nerves and still-existing hair cells is needed, the problem is more straightforward, and promising trends in this direction are being seen. Along with the anatomical studies that reached a similar conclusion (10), our physiological study indicates cochlear synaptopathy is likely to be relatively common, including those with substantial hearing loss, so that regenerative therapies targeting neural regrowth have a strong prospect of finding a ready substrate for reinnervation.

## Conclusion

Cochlear synaptopathy is loss of synapses while hair cells are intact. Though largely accepted to happen in animals, evidence in humans is still limited. We used a combination of deep learning and mathematical modeling to analyze the contributions of the ANN and CM in ECochG. We showed that human subjects-both with and without cochlear implants-are not significantly different from gerbils who have been treated with neurotoxin, indicating some degree of cochlear synaptopathy in these subjects.

## Data availability statement

The raw data supporting the conclusions of this article will be made available by the authors, without undue reservation.

## Ethics statement

The studies involving human participants were reviewed and approved by Institutional Review Boards at the University of North Carolina at Chapel Hill and the Ohio State University. Written informed consent to participate in this study was provided by the participants or legal guardian if less than 18 years old and child’s assent if > 7 years old. The animal study was reviewed and approved by Institutional Animal Care and Use Committee (IACUC) at the University of North Carolina at Chapel Hill.

## Author contributions

RH and DF: conceptualization, software, formal analysis, data curation, writing – original draft, and visualization. RH, KH, WR, KB, HP, OA, and CB: methodology. KH, DF, WR, KB, HP, OA, and CB: investigation. WR, KH, KB, HP, OA, and CB: writing – review and editing. DF: supervision, project administration, and funding acquisition. All authors contributed to the article and approved the submitted version.

## Funding

This work was supported by the Office of the Assistant Secretary of Defense for Health Affairs through the Hearing Restoration Research Program under Award No. W81XWH-19-1-0609. The opinions, interpretations, conclusions, and recommendations are those of the authors and are not necessarily endorsed by the Department of Defense.

## Conflict of interest

DF, OA, CB, HP, and KB have consulting arrangements and research projects with MED-EL, Cochlear Corp, and/or Advanced Bionics. CB also consults for Envoy Medical and IotaMotion. OA and CB have an equity interest in Advanced Cochlear Diagnostics.

The remaining authors declare that the research was conducted in the absence of any commercial or financial relationships that could be construed as a potential conflict of interest.

## Publisher’s note

All claims expressed in this article are solely those of the authors and do not necessarily represent those of their affiliated organizations, or those of the publisher, the editors and the reviewers. Any product that may be evaluated in this article, or claim that may be made by its manufacturer, is not guaranteed or endorsed by the publisher.
